# Detecting continuous structural heterogeneity in single molecule localization microscopy data with a point cloud variational auto-encoder

**DOI:** 10.1038/s41598-025-31201-z

**Published:** 2025-12-03

**Authors:** Sobhan Haghparast, Yi Zhang, Qian Tao, Sjoerd Stallinga, Bernd Rieger

**Affiliations:** Department of Imaging Physics, Delft University of Technology, Delft, 2628 The Netherlands

**Keywords:** Structural biology, Fluorescence imaging, Super-resolution microscopy, Computational science, Imaging techniques

## Abstract

The low degree of labeling and limited photon count of fluorescent emitters in single molecule localization microscopy results in poor quality images of macro-molecular complexes. Particle fusion provides a single reconstruction with high signal-to-noise ratio by combining many single molecule localization microscopy images of the same structure. The underlying assumption of homogeneity is not always valid, heterogeneity can arise due to geometrical shape variations or distinct conformational states. We introduce a Point Cloud Variational Auto-Encoder that works directly on 2D and 3D localization data, to detect multiple modes of variation in such datasets. The computing time is on the order of a few minutes, enabled by the linear scaling with dataset size, and fast network training in just four epochs. The use of lists of localization data instead of pixelated images leads to just minor differences in computational burden between 2D and 3D cases. With the proposed method, we detected radius variation in 2D Nuclear Pore Complex data, height variations in 3D DNA origami tetrahedron data, and both radius and height variations in 3D Nuclear Pore Complex data. In all cases, the detected variations were on the few nanometer scale.

## Introduction

Single Molecule Localization Microscopy (SMLM) is a widely applied super resolution microscopy technique that enables below diffraction limit imaging^1–4^. The resolution of SMLM is mainly limited by the localization precision and the degree of labeling to values typically between 10 and 50 nm^5^. If multiple identical copies of a macromolecular structure are present in an image, the resolution can be improved by fusing these individual structures into a unified structure with increased signal-to-noise ratio^6–9^. Such methods are based on the assumption of homogeneity of the underlying data, that is, it is assumed that all imaged particles are structurally fully identical. This homogeneity assumption, however, does not necessarily hold, not even for chemically identical structures. Heterogeneity in the set of to-be fused particles can originate from biological changes^10^ or sample preparation^11^ and can be continuous or in distinct classes^12^. In Cryogenic Electron Microscopy (Cryo-EM)^13,14^ heterogeneity detection of distinct classes is commonly applied^15–17^. SMLM data are point sets (coordinates of all localization events) that thus differ from pixelated images of Cryo-EM. Moreover, the complex photo-physics of the molecular on-off switching leads to SMLM data that often come with repeated localizations of the same emitter such that the number of localizations is not a linear representation of the actual density of fluorophores^18,19^. In detail, the underlying structure of the particle in SMLM is formed by the set of fluorophore binding sites. These are then stochastically labeled with fluorophores with a certain labeling efficiency (Degree Of Labeling, DOL, equal to the ratio of fluorophore occupation to available binding sites). The fluorophores randomly blink or bind, giving a random number of localization events per fluorophore, determined by the photo-physics. The aggregate of all localization events per particle is the input for the particle fusion algorithm. These differences in acquisition and data between SMLM and Cryo-EM stand in the way of direct application of Cryo-EM methods for particle fusion and for detecting heterogeneity in the underlying dataset, such as the use of learning-based mixed-dimensional Gaussian mixture models^20^.

Detecting distinct heterogeneity in SMLM data can be performed using template-free clustering approaches such as done by Huijben et al.^12^. Such methods, however, cannot be used to identify continuous variations such as geometric shape parameters of the imaged macromolecular structure. Recently, a template-free continuous structural heterogeneity detection method has been proposed by us^21^. A key limitation of the method is its computational complexity, which scales as $N^2$, with *N* the number of particles. In practice, the dataset size is therefore limited to a few hundred particles, which can be analyzed in a few hours. In addition, the ability to detect multiple modes of variation and non-geometrical modes of variation, such as the Degree Of Labeling (DOL) is limited. Alternative approaches for detecting continuous structural heterogeneity include methods with varying requirements and limitations. For example, a proposed learning-based method^22^ is heavily dependent on manual annotation during the training phase. This process is not only time-consuming, but is also prone to human error, and has poor scalability for large datasets. Similarly, the statistical pattern recognition method ECLiPSE^23^ requires precise segmentation and a high signal-to-noise ratio to operate optimally, which limits its effectiveness in scenarios with low DOL or complex environments. LocMoFit^24^, a model-based fitting technique, utilizes a parametric geometric model based on prior knowledge, constraining its adaptability to cases where this prior knowledge is available and reliable.

Here, we propose to overcome these limitations by applying a Variational Auto-Encoder (VAE) to extract generative features from the SMLM dataset, and thereby identifying continuous modes of variation. The VAE^25^ is a probabilistic generative model designed to map the input data, the SMLM point dataset, to a lower-dimensional latent space while preserving the essential statistical properties of the data distribution. Unlike traditional autoencoders that directly learn a deterministic encoding-decoding mapping, VAEs learn a probabilistic mapping by encoding inputs into distributions over latent variables, creating a continuous and smooth latent space where similar data points are mapped to nearby regions. This continuous nature of the latent representation enables interpolation between different data points (and the generation of new samples). In the context of SMLM data, the continuous latent space makes VAEs particularly suitable for characterizing and detecting heterogeneous spatial distributions, as the continuous latent space naturally captures the gradual variations in particle clustering patterns. Application of the VAE neural network architecture directly to the point datasets of SMLM, rather than to pixelated datasets, provides a major step to lower memory requirements and processing speed. Moreover, it provides access to multiple modes of variation via the different latent space dimensions, and is fully template free, minimizing the need for human interaction and avoiding user bias.

In view of the specific application to point clouds, we will refer to the proposed method as Point Cloud Variational Auto-Encoder (PC-VAE) in the following. We evaluated our PC-VAE network on multiple experimental datasets with different numbers of particles and structures, namely: Nuclear Pore Complex (NPC) data imaged in 2D and 3D with Stochastical Optical Reconstruction Microscopy (STORM)^26^, and DNA origami tetrahedrons imaged in 3D with DNA Points Accumulation for Imaging in Nanoscale Topography (DNA-PAINT)^27^.

## Methods

### Point cloud variational auto-encoder framework (PC-VAE)

The purpose of applying VAE to SMLM data is to learn a continuous latent representation of the point cloud that captures the spatial heterogeneity inherent in the particles. The training process leverages a mini-batch-based stochastic gradient descent (SGD) strategy: In each iteration, a mini-batch of *M* particles is sampled, and the model computes the loss per particle, which combines reconstruction accuracy and latent space regularization. The loss, averaged across the *M* particles in the mini-batch, is used to update the model parameters via backpropagation. The optimization process is conducted in multiple epochs, ensuring that the entire dataset is traversed multiple times. As a result, the latent space induced by the trained encoder can effectively capture the spatial diversity and heterogeneity across all particles in the dataset. After training, the model encodes each particle into its respective latent representation, effectively capturing its spatial features for downstream tasks of identifying the structural heterogeneity.

To illustrate the model’s functionality, we take a single particle with *N* localizations as an example. Such a particle with *N* localizations is denoted as a matrix $\textbf{X} = \left[ \textbf{x}_1, \ldots , \textbf{x}_N\right] \in \mathbb {R}^{N \times p}$, with $p=2,3$ the spatial dimension of the dataset. The VAE learns a latent representation $\textbf{z}$ in a lower-dimensional space $\mathbb {R}^d$ ($d \ll N$) through an encoder network $q_\phi (\textbf{z}|\textbf{X})$ and a decoder network $p_\theta (\textbf{X}|\textbf{z})$, where $\phi$ and $\theta$ are the learnable parameters of the encoder and decoder. The encoder maps the input $\textbf{X}$ to a multivariate Gaussian distribution in the latent space, which can be described by its mean vector $\mathbf {\mu }$ and standard deviation $\mathbf {\sigma }$, from which we use the reparameterization trick^25^ to obtain the latent representation of $\textbf{X}$. The reparameterization trick is a technique for making the sampling process differentiable, which is essential for training the VAE using backpropagation:

1 $$\begin{aligned} \begin{aligned} \mathbf {\mu }, \mathbf {\sigma }&= f_\phi (\textbf{X}) \\ \textbf{z}&= \mathbf {\mu } + \mathbf {\sigma } \odot \mathbf {\epsilon }, \end{aligned} \end{aligned}$$

where $f_\phi$ is the forward operation of the encoder neural network, $\odot$ denotes the element-wise product, and $\quad \mathbf {\epsilon } \sim \mathscr {N}(0, \textbf{I})$. With the reparametrization trick, gradients can be computed with respect to the encoder parameters $\phi$, enabling end-to-end training of the VAE using backpropagation. The decoder network $p_\theta (\textbf{X}|\textbf{z})$ aims to reconstruct the cloud of localization points from its latent sample representation. Given a latent vector $\textbf{z} \in \mathbb {R}^d$, the decoder learns to generate a matrix of spatial coordinates $\hat{\textbf{X}} = [\hat{\textbf{x}}_1, \ldots , \hat{\textbf{x}}_N] \in \mathbb {R}^{N \times p}$ that follows the original localizations.

To effectively encode and reconstruct the point cloud, we need to design a neural network architecture that can handle the properties of point clouds, which can be considered as sets. The key property we need for processing point cloud data is permutation invariance, as the ordering of points in a point cloud should not affect its representation. Formally, for any permutation matrix $\textbf{P} \in \{0,1\}^{N \times N}$, it satisfies:

2 $$\begin{aligned} f_\phi (\textbf{P}\textbf{X}) = f_\phi (\textbf{X})\,. \end{aligned}$$

This property is crucial for SMLM data since localizations are inherently orderless and any meaningful feature extraction should be invariant to their input ordering. To achieve this permutation invariance, we adopt a PointNet-based architecture^28^ for our encoder network, which processes each point cloud independently and then aggregates information through symmetric operations. Specifically, in our implementation, we employ three layers of shared-weight multilayer perceptrons (MLPs) with output dimensions of 64, 128, and 1024 to transform each *p*-dimensional localization (coordinates) into a higher-dimensional feature space. This transformation enables the network to capture more complex spatial relationships within the point cloud. Each MLP layer is followed by batch normalization (BN) and rectifier linear unit (ReLU) activation functions. The use of identical MLP weights when processing each localization ensures that the output remains unchanged regardless of how the input localizations are ordered. This architectural design directly enforces permutation invariance in point cloud processing. We further apply mean pooling as the final symmetric aggregation function to get the latent representation vector. This differs from the original PointNet architecture^28^, which uses max pooling for classification and segmentation tasks. Our choice of mean pooling is motivated by the generative nature of our task. While max pooling selectively preserves the most prominent features useful for discrimination tasks, mean pooling retains channel-wise average spatial information for robust latent representation and reconstruction.

For the VAE, the dimensionality of the compressed representation needs manual selection. This often involves a balance between capturing essential variability in the data while maintaining computational efficiency and interpretability. Although there is no universally optimal number of latent dimensions, the choice is guided by empirical testing. The criterion of dimensionality selection is to ensure that the model can effectively capture key generative features without overfitting to noise or irrelevant details. Through iterative evaluation, it is possible to identify a latent space size that balances these factors, enabling the model to capture the relevant modes of variation while avoiding redundancy of latent dimensions. For our architecture, the number of latent dimensions is empirically selected to $d=8$ based on this criterion.

**Fig. 1:**
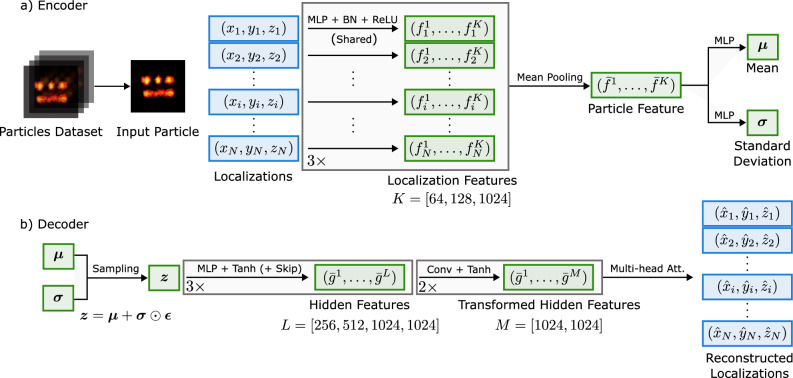
An overview of the proposed PC-VAE framework. For the sake of simplicity, we show the processing steps for a single particle only. The loss function is computed by averaging over a mini-batch of *M* sampled particles. In the encoder, each input localization point is transformed independently through three parallel MLP blocks to higher dimensions. These MLP blocks are applied to each localization individually, meaning that every localization undergoes the same transformation regardless of its position in the sequence. The resulting features are pooled to generate a global particle representation, which is then passed through two separate multi-layer MLPs to produce Gaussian latent variables. The encoder is designed to be invariant to the order of the input points. In the decoder, samples drawn from the Gaussian distribution are transformed to match the shape of the input localizations. In the figure, Skip denotes the skip connection, and Multi-head Att. denotes the multi-head attention mechanism. The reconstruction loss function also ensures that the order of the reconstructed points is irrelevant, maintaining the framework’s robustness to input permutations.

Our proposed decoder network progressively reconstructs the coordinates through a series of expanding transformations. The network first expands the latent vector through fully connected MLPs (256, 512, and 1024 neurons) with hyperbolic tangent (Tanh) activations and batch normalization. We choose the Tanh activations in the decoder since its bounded output range ($-1$ to 1) naturally suits the generation of normalized point coordinates during reconstruction. A skip connection is used at the 1024 neuron layer for direct information flow. The skip connection allows information to bypass intermediate layers and flow directly from an earlier layer to a later one for stable backpropagation during training^29^. The network then applies two 1024 channel localized feature transformations through 1D convolutions, followed by a 4-headed self-attention^30^ that captures global dependencies between different regions of the point cloud. Finally, the network outputs the spatial coordinates of the reconstructed particles. This architecture enables the decoder to reconstruct both the overall structure and the local density variations characteristic of the SMLM data. An overview of the pipeline is shown in Fig. 1.

### Loss function

The loss function of our proposed method consists of two components that balance reconstruction quality and latent space regularization:

3 $$\begin{aligned} \mathscr {L}_{\text {total}} = \mathscr {L}_{\text {recon}} + \beta \mathscr {L}_{\text {KLD}}, \end{aligned}$$

where $\beta \in \mathbb {R}^+$ is the balancing coefficient. To avoid dependence on the ordering of localizations, we measure the quality of the reconstruction using the symmetric Chamfer distance (CD)^31^. The Chamfer distance computes the average minimum distance between points in two sets:

4 $$\begin{aligned} \mathscr {L}_{\text {recon}} = \frac{1}{N} \left( \sum _{\textbf{x} \in \textbf{X}} \min _{\hat{\textbf{x}} \in \hat{\textbf{X}}} |\textbf{x} - \hat{\textbf{x}}|_2^2 + \sum _{\hat{\textbf{x}} \in \hat{\textbf{X}}} \min _{\textbf{x} \in \textbf{X}} |\textbf{x} - \hat{\textbf{x}}|_2^2\right) . \end{aligned}$$

We follow the ordinary use of the Kullback-Leibler divergence (KLD) to regularize the latent space to follow the *d*-dimensional Gaussian distribution which has a closed form expression:

5 $$\begin{aligned} \mathscr {L}_{\text {KLD}} = D_{\text {KL}}(q_\phi (\textbf{z}|\textbf{X})||\mathscr {N}(0, \textbf{I})) = \frac{1}{2} \sum _{i=1}^d (\mu _i^2 + \sigma _i^2 - \log (\sigma _i^2) - 1). \end{aligned}$$

The loss function is computed for each particle in a sampled mini-batch and then averaged over all particles in the mini-batch.

### Training details

We implemented all our network-related codes with PyTorch 2.0^32^ on a workstation with an NVIDIA RTX A40 GPU and an Intel Xeon (3.4 GHz, 4 cores) CPU. We conducted in-house ablation experiments via grid search to determine the hyperparameters for the best model performance and generalization capabilities in our settings. For the results presented here, we used a mini-batch scheme with a batch size of $M=8$. Next, we use the Adam^33^ optimizer with an initial learning rate of $10^{-4}$ and a weight decay of $10^{-5}$. Although VAE is known for its ability to capture the representation of the underlying data, it is also known to be difficult to train with a fixed value of $\beta$^34^. Thus, we used a Sigmoid warm-up of the KLD in the first 5000 optimization steps. The stochastic nature of the VAE training, facilitated by the reparameterization trick, ensures that the learning process effectively captures the underlying data distribution.

### Binning and visualization

To visualize the modes of variations, we ordered the input particles based on each latent unit. This results in 8 different orderings of the particles. The ordered particles are then binned in 20 bins of equal particle number. Next, the particles in each bin are registered together, resulting in a so-called super particle per bin. Then we register the 20 super particles with respect to each other and generate reconstructions per bin for each latent unit. For registration of each point cloud we employed the fast particle fusion approach of Wang et al.^9^. Each super particle then represents a specific state or shape of the particles, ordered according to the bin number.

### Data description

Our proposed algorithm works on datasets consisting of point clouds (*x*, *y*, and depending on the data also, *z* coordinates) derived from SMLM experiments. Particles have been segmented from the overall FOV by manual particle picking software, as used and described earlier^11–13,21^. The resulting datasets typically have a few hundred to a few thousand particles where each particle has typically on the order of hundred localizations. Usually, there are multiple localization events per binding site of the underlying chemical structure. These arise from repeated blinking of a fluorophore that is firmly attached to the binding site (STORM), or from repeated binding/unbinding of a fluorophore that in itself does not blink (PAINT). The dimensionality can be 2D or 3D depending on the image acquisition. Here we use 2D and 3D datasets^21,35^ of Nuclear Pore Complexes (NPCS ), and a DNA origami tetrahedron dataset^21^, with in total 1,399, 3,810 and 218 particles, respectively.

### Model based parameter estimation

In order to verify our PC-VAE approach we need to compare the latent space ordering with independent estimates for the different parameters such as height and radius. As these parameters are not known for experimental data we employ a model based approach described here. For the experimental 2D NPC dataset, we estimate the radius as follows. All particles are centered by subtracting the mean localization from all localizations of a particle, as described in Heydarian et al.^7^. The localization coordinates are then transformed into polar coordinates, and the mean of the radial coordinate is taken as the estimated NPC radius.

For the experimental 3D DNA-origami tetrahedron dataset, we compare the latent space coordinate to the estimated height of the tetrahedron structure. Particles are projected onto the *z*-axis to generate a histogram of *z*-coordinates. This histogram exhibits two peaks, corresponding to the three binding sites at the base plate of the tetrahedron and the top of the tetrahedron, respectively. A mixture of two Gaussian distributions is fitted to this histogram, and the height is calculated as the difference between the means of the two fitted Gaussian distributions.

For the 3D NPC dataset, we detect both the radius and height of the structure. To estimate the radius, the particles are centered as in the 2D case, and the median of the radial coordinate in polar space is used. To determine the height, all localizations are projected onto the *z*-axis. By analyzing the *z*-coordinate histogram, height is estimated as the difference between the median of projected points above and below the central plane defined by the mean of all particles.

To evaluate the precision of each parameter estimate, we calculate the full width at half maximum (FWHM) of the respective histogram and divide it by the square root of the number of localizations to determine the standard error of the mean.

## Results

We applied our proposed PC-VAE method on the above mentioned three experimental datasets and measured different modes of variation. Validation of these template-free modes of variation can only be performed by visual inspection or by an independent model. We therefore show and analyze the super particles obtained from registered particles in each bin for all latent dimensions.

### 2D NPC dataset

**Fig. 2:**
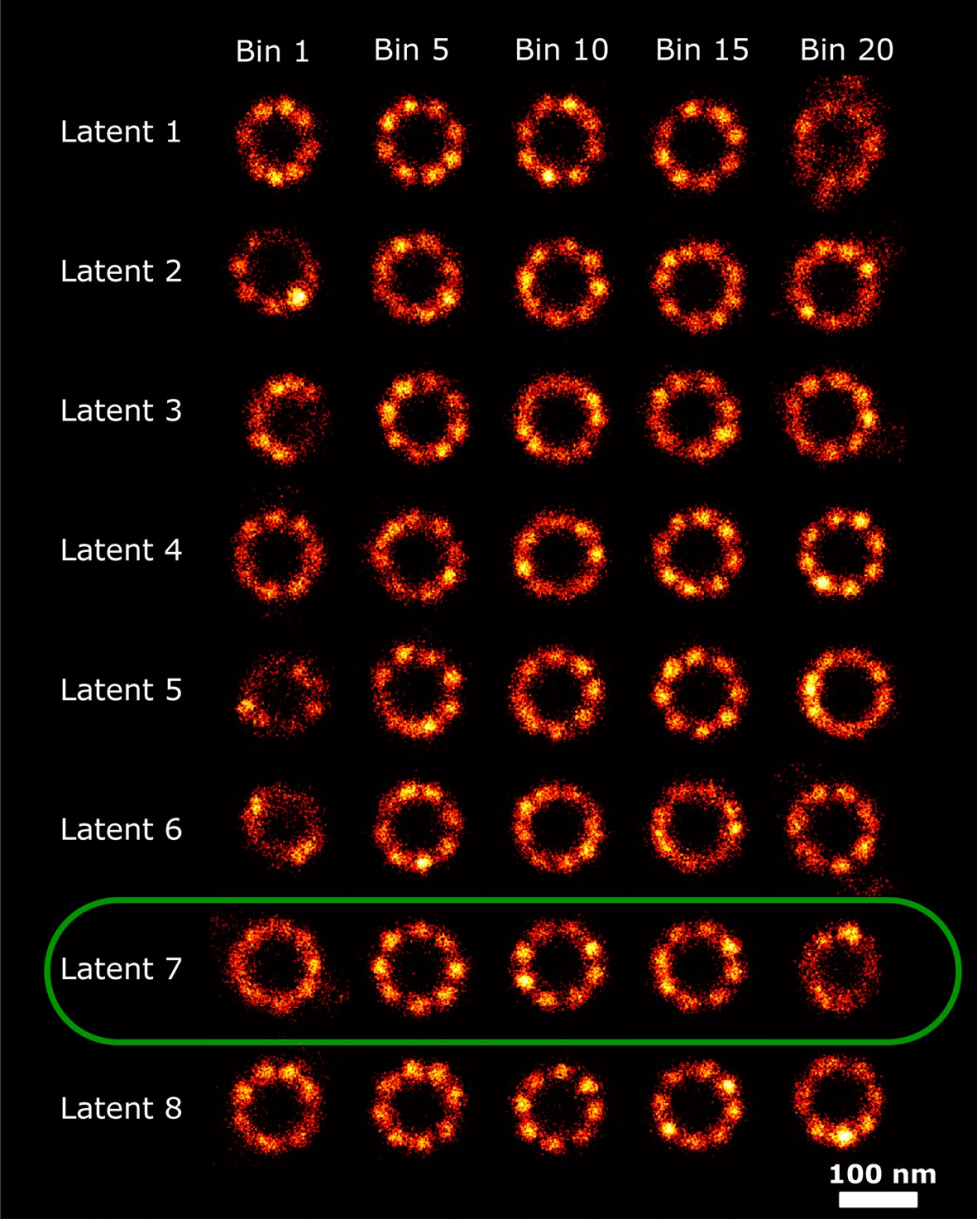
Reconstructions ordered along all latent dimensions for the 2D-NPC dataset. Super-particles reconstructed by registration of particles in 20 bins for all 8 latent space dimensions. For the purpose of concise illustration we only show bins 1, 5, 10, 15 and 20. The reconstructions for latent dimension 7 show continuous heterogeneity in the radius of the NPC ring. The scale bar applies to all images.

**Fig. 3:**
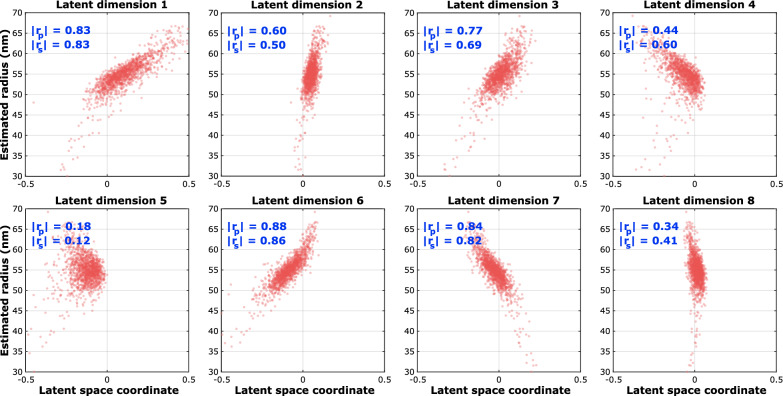
Correlation latent dimensions with radius in the 2D-NPC dataset. Scatter plots of latent coordinate versus estimated radius and computed Pearson and Spearman correlation coefficients.

**Fig. 4:**
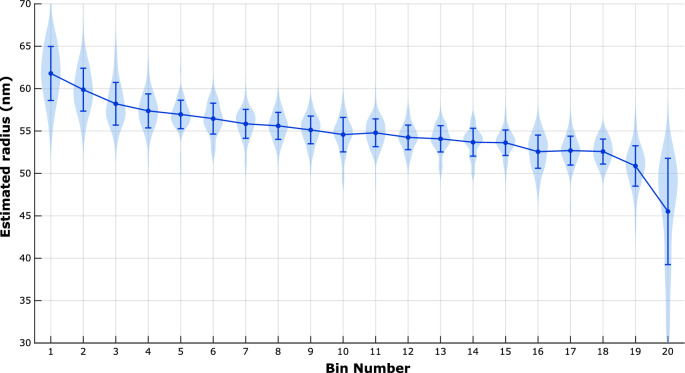
Spread in radius per bin for the 2D-NPC dataset. Violin plots of the distribution of detected radius values within each bin, along with mean and standard deviation.

**Fig. 5:**
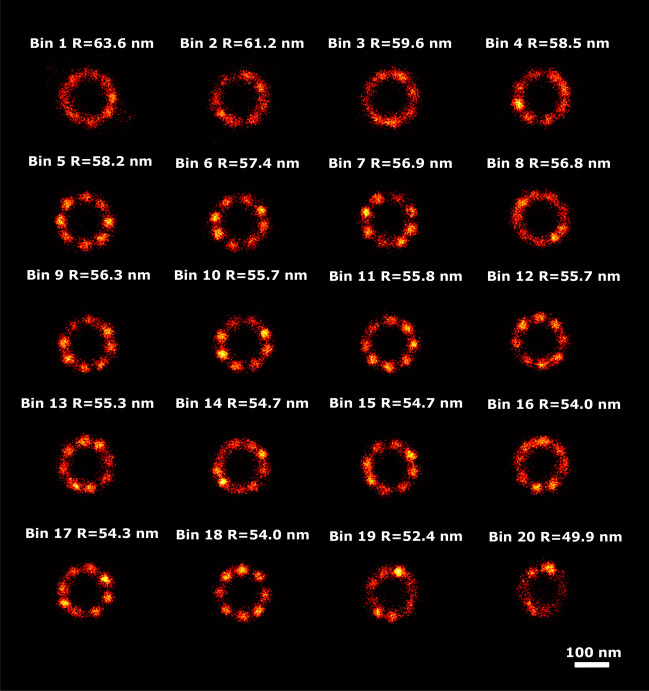
Detected radius variation in the 2D-NPC dataset. Super particles for all 20 bins ordered along latent dimension 7, with estimated radii of the super particles, ranging from 63.6 to 49.9 nm. The uncertainty of the radius estimation is 0.2 nm for all bins. The scale bar applies to all images.

This dataset consists of 1399 particles, with a continuous distribution of ring radii with a mean around 55 nm, and on average 155 localizations per particle. Since each latent space dimension is organized based on the generative features of the data, particles within each bin are considered homogeneous with respect to the generative feature to which the latent space dimension is most sensitive. In Fig. 2 we show super particles for bin number 1, 5, 10, 15 and 20, respectively, in all 8 latent dimensions. Visual inspection of this Figure indicates that latent space dimension 6 and 7 have the clearest correlation with the radius. Other latent dimensions also carry information about the radius as will be explained in the discussion section. We confirmed the selection of the latent dimensions with maximum correlation with radius by estimating the radius of the individual particles and subsequently computing the Pearson and Spearman correlation coefficients of the resulting scatter plots per latent dimension (see Fig. 3). The distribution of estimated radii per bin along latent dimension 7 is shown in Fig. 4, and indicates a spread in radius inside each bin of about 2 to 3 nm, with a total variation in radius of around 15 nm across the latent dimension. Reconstructions per bin are shown in Fig. 5. The detected spread is similar to that found by our earlier continuous heterogeneity detection (CHD) method^21^. The quality of the reconstructions per bin are not improved over those for CHD, which is partly due to a larger number of bins for PC-VAE (20) compared to CHD (10) and hence a smaller number of particles per bin.

**Fig. 6:**
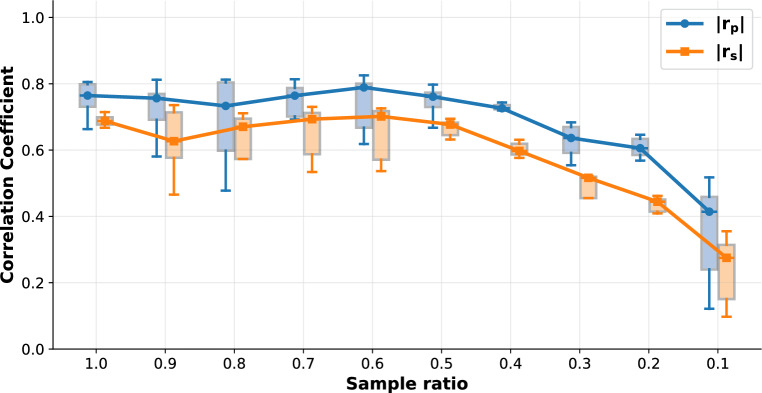
Robustness of latent–radius correlation to missing localizations of the 2D-NPC dataset. Box plots with lines of sample ratios versus absolute Pearson and Spearman correlations. The experiment was implemented by randomly sampling the desired fraction of localizations per particle, while keeping all other experimental settings identical.

We have tested the PC-VAE method for robustness to missing data by randomly deleting fractions of the 2D NPC dataset, running the PC-VAE algorithm, and monitoring the correlation between the latent space dimensions and the NPC radius. Figure 6 shows the dependence of the Pearson and Spearman correlation coefficients for the different dimensions of latent space as a function of sample ratio. It appears that the detected correlations are robust when up to 50% of the data is randomly removed, and only seriously drop when more than about 80% of the data is left out. The average number of localizations per particle in this dataset is 155, giving about 19 localizations on average for each of the 8 binding sites. It follows that the method breaks down when on average there are just a handful of localizations left per binding site. Further reduction would, by statistical fluctuations, make some binding sites fully disappear, which in turn would make the radius correlation undetectable.

### 3D tetrahedron dataset

**Fig. 7:**
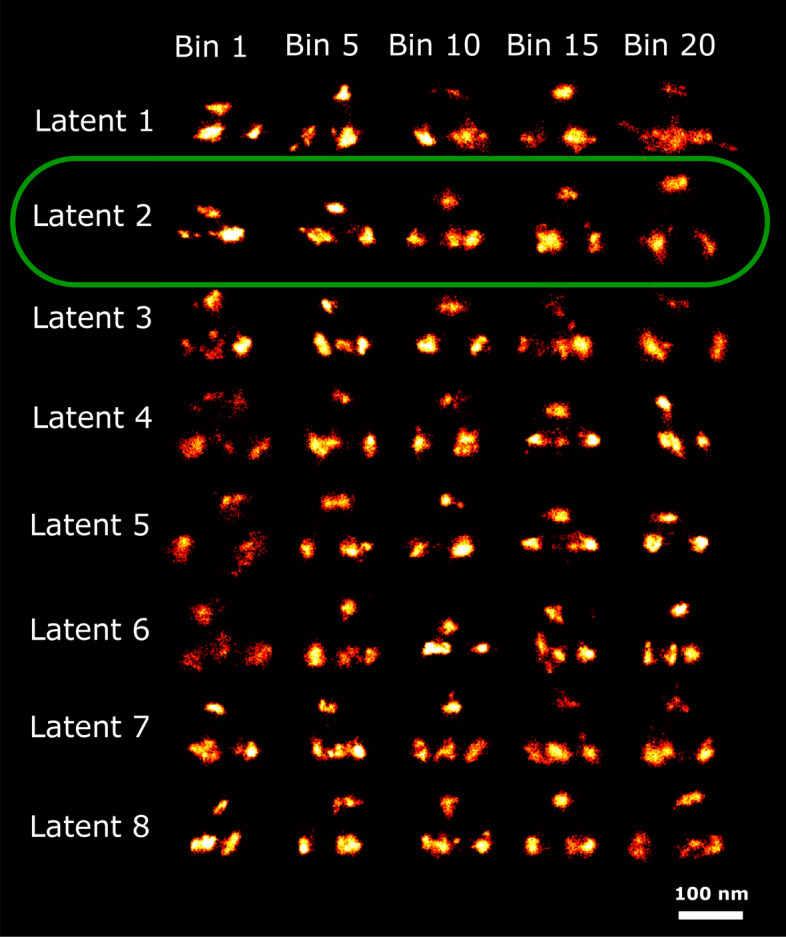
Reconstructions ordered along all latent dimensions of the 3D tetrahedron dataset. Super-particles reconstructed by registration of particles in 20 bins for all 8 latent space dimensions. For the purpose of concise illustration we only show bins 1, 5, 10, 15 and 20. The reconstructions for latent dimension 2 show continuous heterogeneity in the height of the tetrahedrons. The scale bar applies to all images.

**Fig. 8:**
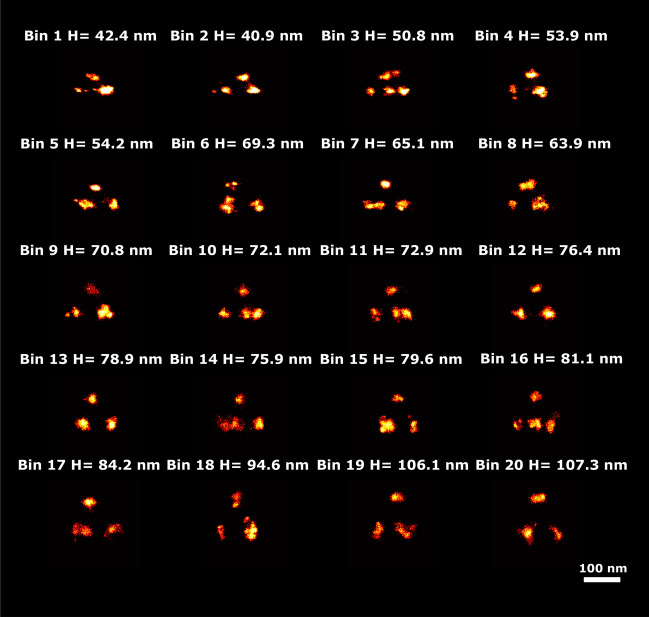
Detected height variation in the 3D tetrahedron dataset. Super particles for all 20 bins ordered along latent dimension 2, with estimated heights, ranging from 42.4 nm to 107.3 nm. The average model estimation precision is around 0.1 nm for all bins. The scale bar applies to all images.

This dataset consists of 218 particles with 4 binding sites per structure. The average number of localizations per particle is 5188, giving over a 1000 localizations per binding site, which is typical for PAINT imaging. Figure 7 shows super particles per bin of just 11 particles (20 bins in total, 20th bin has 9 particles) ordered along the 8 latent dimensions. We manually selected latent dimension 2 for analyzing the relation of this latent dimension to tetrahedron height (see Fig. 8). The PC-VAE framework is able to detect continuous changes in height in 20 bins ranging from 42.4 to 107.3 nm. The variation of height in this dataset was initially reported in three distinct classes (45 nm, 65 nm, 95 nm) by Huijben et al.^12^. Later, the height variation in this dataset was analyzed by us with CHD^21^ in ten bins from 60 to 107 nm , indicating that PC-VAE is able to detect a somewhat broader range of height values. Again the quality of the reconstructions per bin are not improved over those for CHD, due to the same reason as above for the 2D NPC data set.

### 3D NPC dataset

This dataset consists of 3810 cylindrically shaped 3D NPC structures imaged with 3D STORM. Each structure has 32 binding sites in two rings and the average number of localizations per particle is around 80. Recently, this dataset was analyzed by Wang et al.^35^ and compared against an electron microscopy derived representation. This study revealed that the distance between the two rings of the NPC is approximately 48.5 nm, with each ring having a radius of around 55 nm. While variations in radius are expected, they have not been directly measured with light microscopy. Additionally, a 5 nm height difference has been observed between in situ NPCs in HeLa and HEK cells, which could correspond to distinct functional states, such as dilation of the inner ring^36,37^. Our recent CHD approach^21^ is not well-suited for such large datasets, in view of the scaling of the computational complexity with the square of the number of particles. Furthermore, the CHD method struggles with datasets that have a low Degree Of Labeling (DOL), particularly when dealing with complex datasets that include multiple modes of variation. As a result, it failed to identify continuous structural heterogeneity in this dataset.

**Fig. 9:**
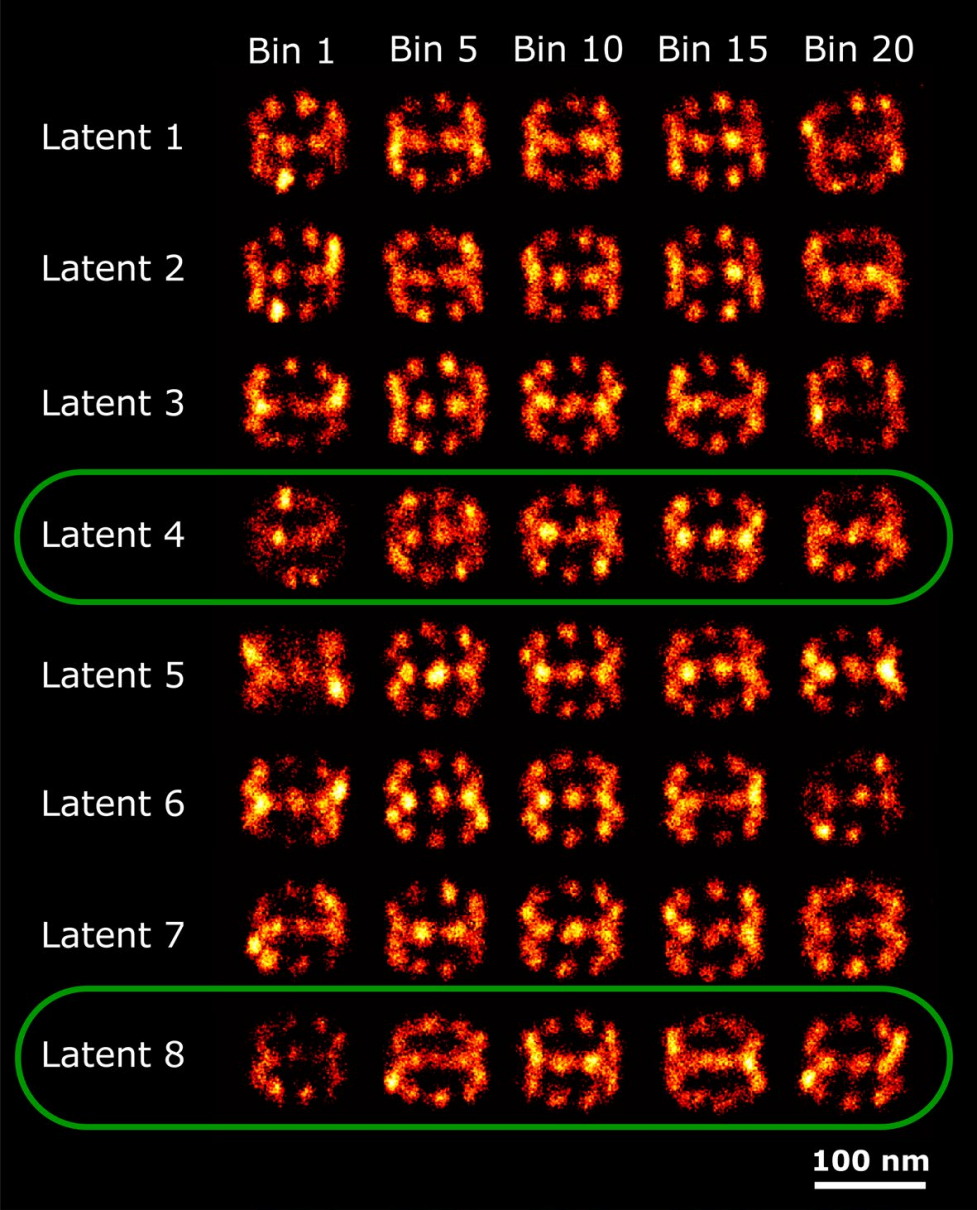
Reconstructions ordered along all latent dimensions of the 3D-NPC dataset. Super-particles reconstructed by registration of particles in 20 bins for all 8 latent space dimensions. For the purpose of concise illustration we only show bins 1, 5, 10, 15 and 20. The reconstructions for latent dimension 4 show continuous heterogeneity in the radius of the NPCs, latent dimension 8 in the height of the NPCs. The scale bar applies to all images.

**Fig. 10:**
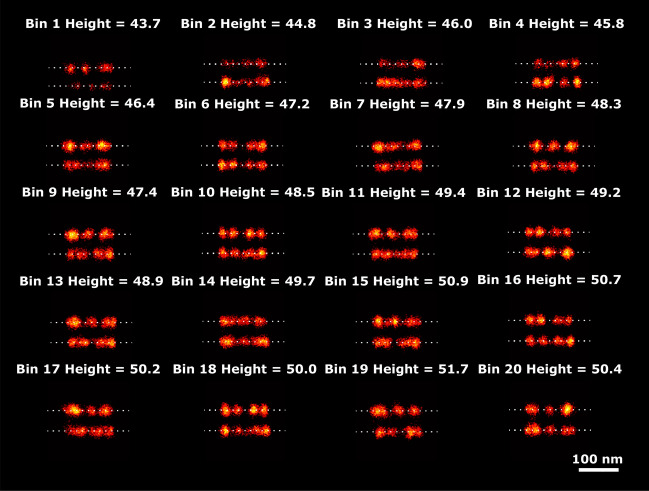
Detected height variation in the 3D NPC dataset. Super particles for all 20 bins ordered along latent dimension 8, with estimated heights, ranging from 43.7 to 51.7 nm. The average model estimation precision is around 0.3 nm for all bins. The scale bar applies to all images.

**Fig. 11:**
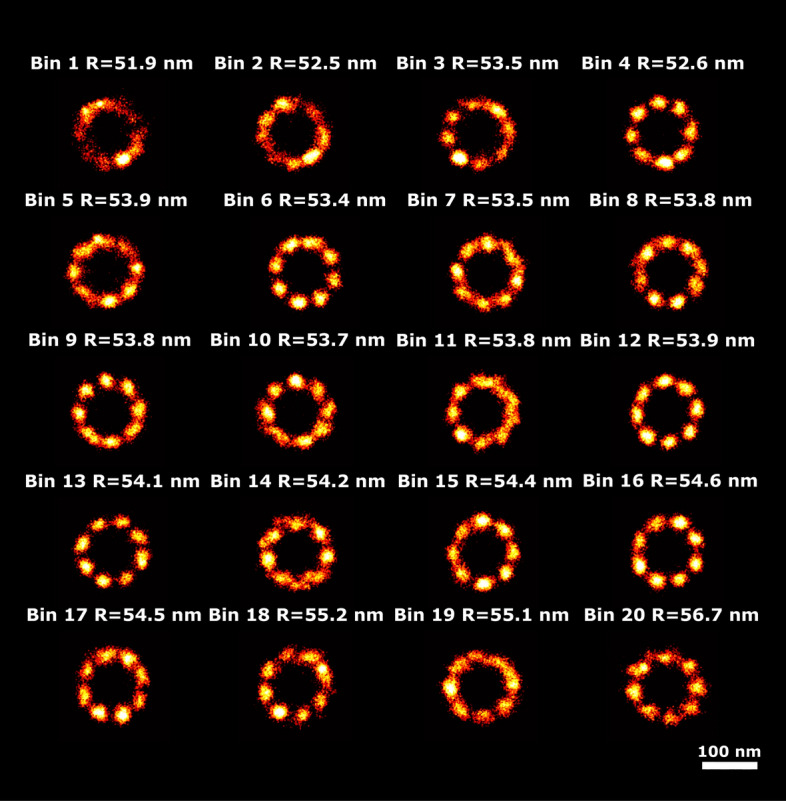
Detected radius variation in the 3D NPC dataset. Super particles for all 20 bins ordered along latent dimension 4, with estimated radii, ranging from 51.9 to 56.7 nm. The average model estimation precision is around 0.2 nm for all bins. The scale bar applies to all images.

Figure 9 shows the reconstructed super particles for all latent dimensions, indicating both radius and height variations in latent dimensions 4 and 8. In Figs. 10 and 11 we show the reconstructions for all 20 bins ordered along those latent dimensions, along with the estimated radius and height. This results in detected continuous heterogeneity in the radius ranging from 51.9 to 56.7 nm and a height variation ranging from 43.7 to 51.7 nm.

## Discussion and outlook

VAEs are commonly used for (lossy) compression of data while retaining the most important data structural components. This happens mainly by detecting the generative features or the most important information to reproduce the data. By mapping the input data into a latent space, VAEs aim to capture the underlying structure in a compressed representation. In this study, we have developed a VAE based on point clouds to capture the low-dimensional representations of SMLM data, with the goal of identifying the inherent continuous structural variations. Our PC-VAE method demonstrates major improvements in computational efficiency compared to the MDS-based CHD approach^21^. First of all, the computational time scales linearly with the number of particles, as opposed to the quadratic scaling of computing the pairwise distance metric in the CHD approach. Second, the PC-VAE method works directly on lists of localizations, which does not lead to significant differences in computational time for 2D and 3D cases. Third, the learning phase of the algorithm converges very quickly, only 4 epochs turned out to be necessary for the cases that were considered. The resulting computing times are very short indeed. The 2D NPC dataset with 1399 particles takes 24 seconds per epoch, the 3D tetrahedron dataset with 218 particles takes 13 seconds per epoch, and the 3D NPC dataset with 3810 particles takes 279 seconds per epoch. This results in typical computing times on the order of a few minutes. The dramatic reduction in processing time of PC-VAE proves the feasibility of scaling to larger datasets without sacrificing performance. Even with this effort, the CHD method was not able to detect height and radius variations because of the lack of sensitivity of the cost function to complex structures such as the NPC in 3D. In comparison to template or model driven approaches PC-VAE, just like CHD, has the advantage of being inherently free of any a priori bias regarding the underlying geometric structure. Fitting to a model or template necessarily confirms the model or template. The vast gain in computational cost of PC-VAE compared to CHD makes it also competitive to template approaches, as these also typically scale linearly with the number of particles. In addition to linear scalability, the fast convergence of PC-VAE also suggests the effectiveness of focusing on coordinates instead of pixelated images, as the network learns directly from the geometrical information that is characteristic of the modes of particles. A full comparison would be worthy of a follow-up study.

**Fig. 12:**
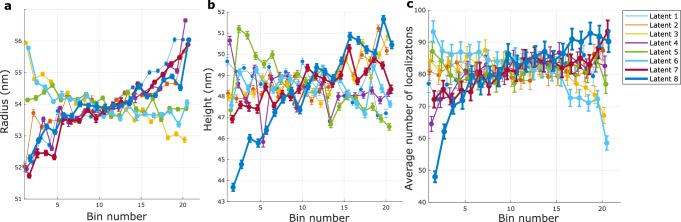
Detected heterogeneity parameters along all latent dimensions for the 3D NPC dataset. This figure illustrates the correlation between each latent dimension and three modes of variations: (**a**) radius, (**b**) height, and (**c**) number of localizations. The legend applies to all panels.

Regarding the disentanglement of different modes of variation, it turns out that the majority of the latent dimensions is correlated with some mode of variation that is present in the data. This makes isolating distinct factors of variation challenging, as the latent space does not naturally separate them into independent components. In Fig. 12 the correlation between each latent space and 3 different modes of variation (radius, height, number of localizations) is plotted for the 3D NPC dataset. Here we used the number of localizations as a rough estimate of the DOL. The first plot indicates that, while many of the latent dimensions are correlated with the change in radius, some exhibit an increasing trend, while others show a decreasing behavior. In particular, in latent dimension number 4, the radius variation is clearly observed. Similarly, the second plot shows increasing and decreasing dependence of height on the latent dimension coordinate for different latent dimensions, and latent dimension 8 showing the most clear trend in the height for each bin. The third plot shows a clear trend with the number of localizations especially for latent dimension 8. The impact of a low and high degree of labeling can also be observed in Fig. 10. Approaches such as the total correlation variational auto encoder (TC-VAE)^38^, which aims to reduce dependencies between latent variables, could be explored to improve the disentanglement of different modes of variation, allowing for more interpretable and controllable latent representations. Another inroad to investigate and improve the heterogeneity disentanglement and quantification is via simulation of a known ground truth^21^.

We foresee two different uses of PC-VAE in an application workflow. In a hypothesis driven workflow where the aim is to disentangle a mode of variation that is a priori foreseen PC-VAE can efficiently order or cluster the particles in a one or low dimensional sub-space of the overall higher dimensional latent space. After such ordering or clustering this may be used to generate better particle fusion reconstruction based on parts of the dataset zooming in on the heterogeneity axis. Also it may be possible to quantitatively map the heterogeneity within the dataset using PC-VAE by retrieving the distribution of particles along the targeted heterogeneity axis. This mapping can be used to support or guide the selection of the axis by maximizing separability along the axis. A second workflow is entirely discovery driven, where no hypothesis is present. In that case the use of PC-VAE is qualitative as the mode of variation has to be identified and then quantified. Selecting a lower dimensional sub-space of the overall latent space for disentangling heterogeneity is then purely based on visual inspection. In case this can be successfully applied one could revert to the first workflow.

The designed PC-VAE can also be adapted to incorporate localization uncertainty, by modifying the Chamfer cost function to account for uncertainties, and so designing a new model that integrates these variations. However, the design and potential convergence of such a model would also need to be carefully considered, as the introduction of uncertainties could affect the stability and efficiency of training. By accounting for potential variations in the data, this approach could offer a more reliable representation by balancing the competing demands of reconstruction accuracy and latent space heterogeneity.

In principle, PC-VAE can incorporate additional quantitative features such as foreground count, background count, or PSF width by appending them to the point coordinates as extra input dimensions. We chose not to do this as variations in these parameters can be expected to correlate to the photo-physical behaviour of the fluorophores and not to the underlying structural variations, while at the same time could be more dominant than the geometric heterogeneity, thus overshadowing the structural variations in the latent space dimensions. In addition, this would also require a significant redesign of the PC-VAE network since these extra features do not have the same physical dimension (and numerical dynamic range) as the localization coordinates. In an exploratory sense it could be interesting to test if and if so what kind of heterogeneity is actually revealed by including these parameters.

Finally, we foresee that PC-VAE can be adapted for a variety of other applications within SMLM, such as image registration and particle tracking, as it is a machine learning approach specifically tailored for SMLM data.

## Data Availability

The single molecule localization data is accessible via the 4TU.research repository at https://data.4tu.nl/private_datasets/dbgcLOAnBsWzZUJjoVbhcXgirKJmP9ycA3FlmfFcJj0.

## Code availability

The used software can be downloaded from https://github.com/Sobhanhaghparast/PCVAE.
